# Diagnostic performance of high and ultra-high-resolution photon counting CT for detection of coronary artery disease in patients evaluated for transcatheter aortic valve implantation

**DOI:** 10.1007/s10554-024-03273-x

**Published:** 2024-11-04

**Authors:** Simran P. Sharma, Sarah Verhemel, Alexander Hirsch, Judith van der Bie, Marcel L. Dijkshoorn, Joost Daemen, Nicolas van Mieghem, Ricardo P. J. Budde

**Affiliations:** 1 Department of Cardiology, Cardiovascular Institute, Thorax Center, Erasmus MC, Rotterdam, The Netherlands; 2 Department of Radiology and Nuclear Medicine, Erasmus MC University Medical Centre, Nd-547, Dr. Molewaterplein 40, 3015 GD, Rotterdam, CA 2040, 3000, The Netherlands

**Keywords:** Computed tomography angiography, Transcatheter aortic valve replacement, Coronary artery disease, Diagnostic imaging

## Abstract

We assessed the diagnostic performance of both ultra-high-resolution (UHR) and high-resolution (HR) modes of photon-counting detector (PCD)-CT within the confines of standard pre-TAVI CT scans, as well as the performance of UHR mode adjusted specifically for coronary imaging, using quantitative coronary angiography (QCA) as the reference. We included 60 patients undergoing pre-TAVI planning CT scans. Patients were divided into 3 groups: 20 scanned in HR mode, 20 in UHR mode, and 20 in adjusted UHR mode, on a dual-source PCD-CT. The adjusted UHR mode employed a lower tube voltage (90 kV vs. 120 kV) and a higher image quality level (65 vs. 34) to enhance coronary artery visualization. Patients underwent invasive coronary angiography as part of clinical routine. CCTA and QCA were reviewed to assess CAD presence defined as stenosis ≥ 50% in proximal and middle coronary segments. We included 60 patients (mean age 79 ± 7 years; 39(65%) men). Mean heart rate during scanning was 72 ± 13 bpm. Median coronary calcium score was 973 [379–2007]. QCA identified significant CAD in 24 patients (40%): 9 patients scanned with HR mode, 10 patients with the UHR mode, and 5 patients with the UHR adjusted mode. Per-patient area under the curves were 0.57 for HR, 0.80 for UHR, and 0.80 for adjusted UHR, with no significant differences between the scan modes, and per-vessel the area under the curves were 0.73 for HR, 0.69 for UHR, and 0.87 for adjusted UHR, with significant differences between UHR and adjusted UHR (*p* = 0.04). UHR and adjusted UHR modes of dual source PCD-CT show potential for improved sensitivity and negative predictive value for detecting CAD in patients undergoing pre-TAVI scans, however, no statistically significant difference from HR mode was observed.

## Introduction

Computed Tomography (CT) is essential in the pre-procedural planning of transcatheter aortic valve implantation (TAVI) [1]. To identify coronary artery disease (CAD), TAVI patients typically also undergo pre-procedural invasive coronary angiography (ICA) [1]. However, by integrating coronary CT angiography (CCTA) with pre-TAVI CT, these patients can benefit from a non-invasive alternative for CAD assessment [2–4]. Despite its potential benefits, the use of CCTA for CAD assessment in TAVI patients remains challenging due to the presence of severe coronary calcifications and stents [1, 5].

Photon-counting detector (PCD)-CT has the potential to overcome the limitations of conventional CT scanners [6, 7]. PCD-CT is equipped with a semiconductor detector instead of the scintillation detector found in conventional scanners [8]. This detector enables the direct conversion of X-ray photons into an electrical signal, facilitating individual measurement of each photon. These detectors are also more geometrically efficient than the conventional energy-integrating detectors increasing spatial resolution with slice thicknesses of 0.4 mm in high-resolution and 0.2 mm slice thickness in ultra-high-resolution (UHR) mode [6, 8–11]. Notably, in a recent study involving patients undergoing pre-TAVI CT scans, including those with severe coronary calcification or prior stent placement, UHR PCD-CT displayed high diagnostic accuracy in detecting CAD [12].

When employing pre-TAVI CT scans for concomitant CAD detection, it is important to acknowledge that the scanning protocols are primarily tailored for aortic valve imaging and not specifically optimized for coronary artery assessment. However, these parameters can be adjusted to specifically target coronary arteries [13]. Therefore, our study aims to investigate the diagnostic performance of both high-resolution (HR) and ultra-high-resolution (UHR) modes of PCD-CT within the confines of standard pre-TAVI CT scans in our institution, as well as evaluate the performance of UHR mode adjusted specifically for coronary imaging, for the assessment of CAD with quantitative coronary angiography (QCA) as the reference standard.

## Methods

### Study population

We evaluated 60 consecutive patients who underwent pre-TAVI CT scans using a dual-source PCD-CT scanner between February 2022 and January 2024. Specifically, 20 patients underwent scanning in the HR mode, 20 in UHR mode, and 20 in UHR mode adjusted for coronary imaging. The exclusion criteria included patients without diagnostic coronary angiography (ICA) and patients who did not provide informed consent. This study was performed in line with the principles of the Declaration of Helsinki. The ethics committee waived full review and approval because of the observational, retrospective nature of the study.

### Scan protocol

All patients were scanned on a dual-source PCD-CT scanner (NAEOTOM Alpha, software version syngo CT VA50; Siemens Healthineers). First, a non-contrast acquisition was performed to determine coronary artery and aortic valve calcification scores. Subsequently, a contrast-enhanced ECG-gated CT of the heart was acquired. In the standard pre-TAVI CT protocol, HR mode employed a 144 × 0.4 mm collimation, while UHR mode employed a 120 × 0.2 mm collimation, both at a tube voltage of 120 kV and an image quality level of 34. Furthermore, an adjusted UHR mode was also employed with a reduced tube voltage of 90 kV and an increased image quality level set to 65. The reconstructed matrix size (512 × 512 pixels, 768 × 768 pixels or 1024 × 1024 pixels) was automatically determined by the scanner. The acquisition protocol also included a high-pitch aortoiliac CT angiography for access route assessment. Detailed parameters of the scan protocol are presented in Table 1. Beta-blockers and nitroglycerin were not administered.

**Table 1:** Scan parameters across pre-TAVI CT protocols

Scan parameter	TAVI protocol HR	TAVI protocol UHR	TAVI protocol adjusted UHR
Scan mode	Prospective full width 5.76 mm	Prospective full width 2.4 mm	Prospective full width 2.4 mm
kVp	120	120	90
Image quality level	34	34	65
Contrast protocol*	45 ml at 3 ml/s	45 ml at 3 ml/s	Test bolus: 15 ml, followed by CTA: 85 ml at 5 ml/s
Scan Timing	Bolus tracking	Bolus tracking	Test bolus technique
ECG gating window	Regular rhythm: 15–45% Irregular rhythm: 150-450ms	Regular rhythm:15–45% Irregular rhythm: 150-450ms	Regular rhythm: 20–85% Irregular rhythm: 150–550 ms.
Slice thickness	0.4 mm	0.2 mm	0.2 mm
Image Kernels	Bv48	Bv48, Bv56, Bv64	Bv48, Bv56, Bv64

* Visipaque 320 mg iodine/mL (Jodixanol), GE Healthcare. HR = high-resolution; UHR = ultra-high-resolution

### Objective image quality assessment

For the objective image quality assessment, the sharpest kernel images (Bv56, quantum iterative reconstruction at level 4) available were used. Regions of interest were placed in the ascending aorta and subcutaneous adipose tissue. The regions of interest were drawn as large as possible to measure the mean CT numbers, expressed in Hounsfield Units, along with their standard deviations (STD). Using these values, the signal-to-noise ratio (SNR) and contrast-to-noise ratio (CNR) were calculated with the following equations:

$$SNR = {{MEA{N_{aorta}}} \over {ST{D_{aorta}}}}\,\,\,and\,\,\,\,CNR = {{MEA{N_{aorta}} - MEA{N_{sub}}} \over {ST{D_{aorta}}}}$$

### Qualitative CAD assessment using CCTA

Qualitative stenosis quantification was conducted by a single radiologist, who was blinded to the invasive coronary angiography and QCA results. The radiologist selected the most appropriate kernel (e.g., Bv48, Bv56, or Bv64) on a case-by-case basis. Significant CAD was defined as a diameter stenosis of ≥ 50% by visual assessment. The assessment was conducted for 9 coronary segments based on the American Heart Association coronary segment classification (segments 1, 2, 3 of the RCA, 5 left main, 6, 7 of the LAD and 11, 12, 13 of the Cx) [14]. Bypass grafts were not included in the analysis. Segments that were scored as non-diagnostic due to compromised image quality were classified as significant stenosis (worst-case scenario). Only segments that were also evaluable on the QCA were included in the analyses. Segments that were non-diagnostic due to size on the CCTA were excluded from the analysis for that vessel. If the remaining segments of that vessel showed no significant stenosis, the vessel was regarded as having no significant stenosis. In this case, only the remaining evaluable segments were assessed on QCA, and the segment that was non-diagnostic due to size on the CCTA was excluded. For the per-patient analysis, if an entire vessel was non-diagnostic due to size, that vessel was excluded from the analysis.

### ICA and QCA

ICA was performed with standard techniques. Two-dimensional QCA analysis was performed using dedicated software (CAAS, version 8.1.1, Pie Medical Imaging, Maastricht, Netherlands). Two orthogonal views were evaluated to measure the maximum vessel stenosis. Angiograms were assessed by two readers who were blinded to the site report results. The primary reader, who underwent specific training in analysis, reviewed each angiogram and subsequently performed QCA on each potentially obstructive lesion. The reviewer manually delineated the straight-line path of the artery across the lesion, which was utilized by the CAAS software to determine the maximum stenosis diameter. A secondary reader, a cardiac physiologist, ensured the quality and accuracy of the QCA. QCA analysis was conducted for the same 9 epicardial segments as for CT. Significant stenosis on QCA was defined as a diameter stenosis of ≥ 50%.

### Statistical analysis

Categorical variables are expressed as frequencies and percentages, continuous variables were expressed as mean ± standard deviation or as median with inter-quartile range, depending on the distribution. Normality was tested by using the Shapiro-Wilk test. For the comparison of normally distributed continuous variables across the three scan modes, the One-Way Analysis of Variance was employed. For continuous variables that were not normally distributed, the Kruskal-Wallis test or the Mann-Whitney U test (for the subgroup analysis) was employed. For the comparison of categorical variables across the three scan modes, the Chi-Square test or Fisher’s Exact test was used, as appropriate. Sensitivity, specificity, positive predictive value, negative predictive value, accuracy, and area under the curve (AUC) were calculated on a patient- and vessel-based level. The AUCs were compared using the DeLong test. A two-tailed p-value of < 0.05 was considered statistically significant. All statistical analyses were performed using SPSS statistical software (IBM Corp. Released 2016. IBM SPSS Statistics for Windows, Version 28.0.1.0 Armonk; NY: IBM Corp.) and R Statistical Software (R version 4.1.1 (2021-08-10)).

## Results

### Study population

A total of 60 patients (mean age 79 ± 7 years; 39 (65%) men) were included. Twelve (20%) patients had ≥ 1 coronary stents implanted, 11 (18%) patients had previously undergone coronary artery bypass graft surgery, and 4 (7%) patients had a pacemaker implantation. The mean heart rate during scanning and the median calcium score did not differ between the three groups (*p* = 0.41; *p* = 0.27). The median volumetric computed tomography dose index (CTDI_vol_) was significantly different across the three scan modes: HR: 11.6 [9.4–15.3] mGy, UHR: 18.6 [14.8–22.2] mGy, and adjusted UHR: 20.5 [18.3–26.9] mGy (*p* < 0.001). The median size-specific dose estimate also showed significant differences: 15.2 [13.2–18.1] mGy, 23.4 [21.2–27.5] mGy, and 25.8 [24.5–34.6] mGy (*p* < 0.001). Similarly, the median dose length product varied significantly: 176 [141-220.5] mGy*cm, 240 [187.3-298.3] mGy*cm, and 306.5 [262.5-419.8] mGy*cm (*p* < 0.001). The patient characteristics and the radiation dose are presented in Table 2.

**Table 2:** Patient demographics and CCTA Radiation exposure

Patient characteristics	Total *N* = 60	TAVI protocol HR	TAVI protocol UHR	TAVI protocol adjusted UHR	*P* value
Male (%)	39 (65%)	12 (60%)	13 (65%)	14 (70%)	0.80
Age, years	79 ± 7	78 ± 8	79 ± 7	80 ± 7	0.62
BMI, kg/m^2^	27 [24–30]	27 [24–30]	29 [25–31]	25 [23–27]	0.12
Prior PCI with stent placement (%)	12 (20%)	6 (30%)	4 (20%)	2 (10%)	0.29
Prior coronary artery bypass graft surgery (%)	11 (18%)	5 (25%)	2 (18%)	4 (36%)	0.46
Heart rate (beats per minute)	72 ± 13	71 ± 14	69 ± 13	75 ± 13	0.41
Median coronary calcium score	973 [379–2007]	973 [411–1661]	1896 [815–2738]	841 [193–2007]	0.27
Median aortic valve calcium score	2815 [2050–3896]	2816 [1752–4193]	2859 [2459–3610]	2598 [1843–3701]	0.84
**CCTA radiation dose**					
CTDI_vol_, mGy*	18 [13–22]	12 [9–15]	19 [15–22]	20 [18–27]	< 0.001
SSDE, mGy*	23 [18–28]	15 [13–18]	23 [21–27]	26 [25–35]	< 0.001
DLP, mGy*cm*	244 [187–312]	176 [141–221]	240 [187–298]	307 [263–420]	< 0.001
**Objective image quality**					
SNR**	7 [6–9]	8 [7–11]	6 [6–8]	7 [6–8]	0.017
CNR**	9 [8–11]	11 [9–14]	9 [8–11]	8 [7–10]	0.003

Data is presented as mean ± Standard Deviation (SD), median [25th – 75th percentile], or frequencies (percentage). CCTA = coronary computed tomography angiography; DLP = dose-length product; HR = high resolution; PCI = percutaneous coronary intervention; CTDIvol = Volumetric computed tomography dose index; SSDE = size-specific dose estimate; UHR = ultra-high-resolution.* Subsequent statistical analyses indicated significant differences in CTDI_vol_ between the scanning modes: UHR vs. HR (*p* < 0.001), adjusted UHR vs. HR (*p* < 0.001), UHR vs. adjusted UHR (*p* = 0.049). For SSDE there were significant differences between the scanning modes: UHR vs. HR (*p* < 0.001), adjusted UHR vs. HR (*P* < 0.001), and for UHR vs. adjusted UHR (*p* = 0.018). For DLP there were significant differences between the scanning modes: UHR vs. HR (*p* = 0.006) and adjusted UHR vs. HR (*P* < 0 0.001) and for UHR vs. adjusted UHR (*p* = 0.003). ** Subsequent statistical analyses indicated significant differences in SNR between the scanning modes: UHR vs. HR (*p* = 0.016) and adjusted UHR vs. HR (*p* = 0.012), but not for UHR vs. adjusted UHR (*p* = 0.85). For CNR there were significant differences between the scanning modes: UHR vs. HR (*p* = 0.023) and adjusted UHR vs. HR (*P* < 0.001), but not for UHR vs. adjusted UHR (*p* = 0.29)

**Fig. 1:**
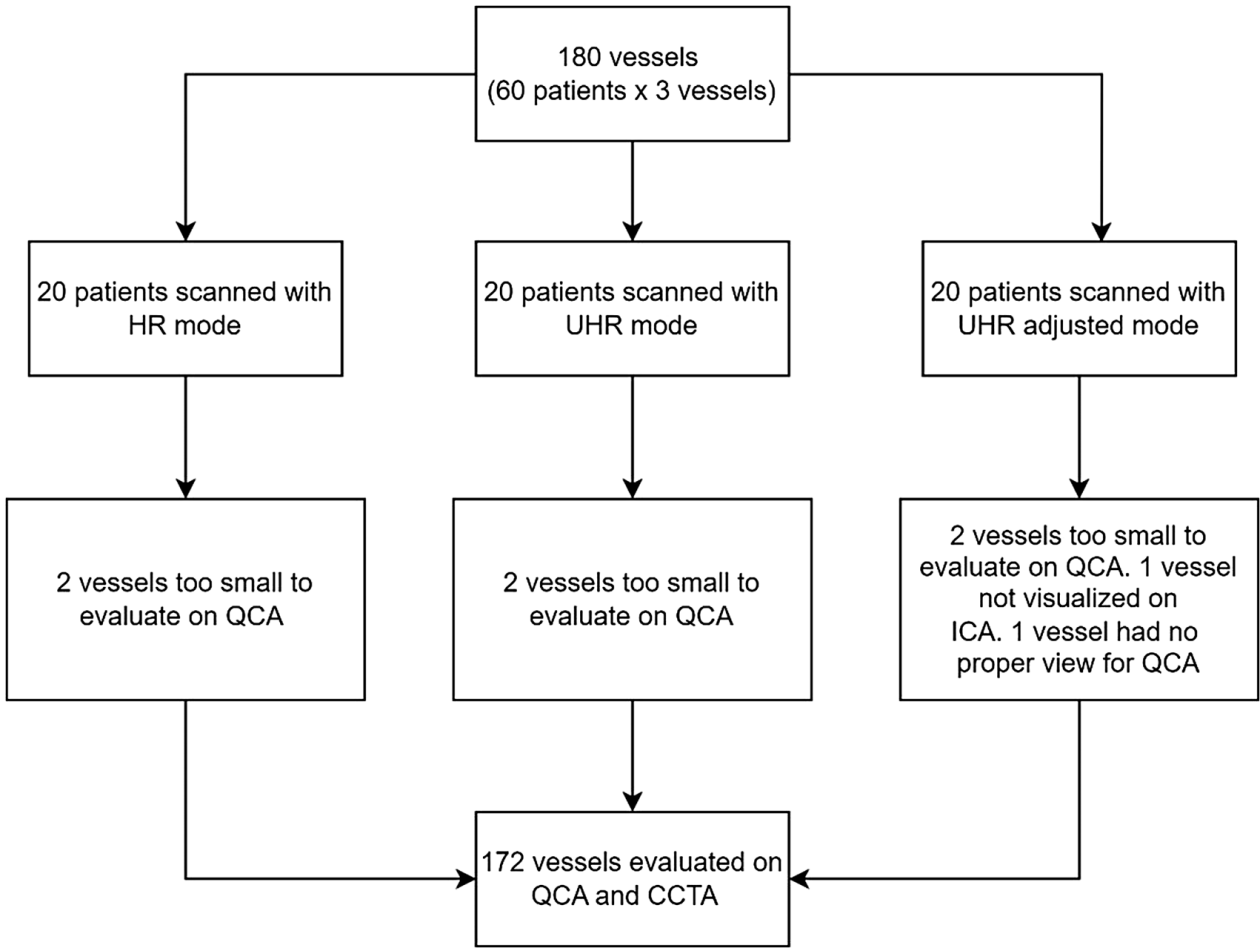
Patient inclusion flowchart

### Image quality assessment and diagnostic performance of CCTA for CAD assessment

Of the potential 180 coronary vessels (60 patients x 3 vessels), 8 (4%) vessels were non-evaluable on QCA. Specifically, 6 vessels were too small to evaluate, 1 vessel was not visualized on the ICA and 1 vessel had no proper view to perform the QCA (Fig. 1). In the objective assessment of image quality, the median SNR values were significantly different across the three scan modes: HR: 8.5 [6.8–11.1], UHR: 6.4 [5.6-8.0], and adjusted UHR: 7.2 [5.9–8.1] (p value: 0.017). The median CNR values were also different across the three scan modes: HR: 11.1 [8.9–14.2], UHR: 8.9 [8.0-11.4], and adjusted UHR: 8.5 [7.3–9.9] (*p* = 0.003) (Table 2). The significant differences were primarily driven by the comparisons of UHR vs. HR (SNR: *p* = 0.016; CNR: *p* = 0.023) and adjusted UHR vs. HR (SNR: *p* = 0.012; CNR: *p* < 0.001).

Twenty-four (40%) patients had a significant stenosis on QCA. Of the 172 evaluated vessels 31 (18%) vessels had a significant stenosis on QCA. Overall, CCTA correctly ruled out CAD in 19 of the 36 patients. In the per-patient analysis, the sensitivity was 100% for both UHR and adjusted UHR modes, while the HR mode demonstrated a sensitivity of 78%. The AUC for UHR and adjusted UHR was both 0.80 and 0.57 for HR and these AUCs were not significantly different.

In the per-vessel analysis, the sensitivity for the adjusted UHR mode remained 100%, but dropped to 67% for the UHR mode and increased to 83% for the HR mode. The adjusted UHR mode demonstrated the highest AUC of 0.87. However, no statistically significant difference in AUC was observed between the UHR mode and HR mode, or between the HR mode and the adjusted UHR mode. There was a significant difference in AUC between the UHR mode and the adjusted UHR mode (*p* = 0.04). A detailed overview of the diagnostic performance of the scanning protocols for the detection of CAD is provided in Table 3.

**Table 3:** Diagnostic performance of HR, UHR and adjusted UHR scanning for the detection of coronary artery disease

.	HR mode	UHR mode	adjusted UHR mode
Per patient	*N* = 20	*N* = 20	*N* = 20
Sensitivity (%)	78 (40–97)	100 (69–100)	100 (48–100)
Specificity (%)	36 (11–69)	60 (26–88)	60 (32–84)
NPV (%)	67 (22–96)	100 (54–100)	100 (66–100)
PPV (%)	50 (23–77)	71 (42–92)	45 (17–77)
Diagnostic accuracy (%)	55 (32–77)	80 (56–94)	70 (46–88)
AUC*	0.57 (0.36–0.78)	0.80 (0.64–0.96)	0.80 (0.67–0.93)
**Per vessel**	***N = 58***	***N = 58***	***N = 56***
Sensitivity (%)	83 (52–98)	67 (35–90)	100 (59–100)
Specificity (%)	63 (48–77)	72 (57–84)	73 (59–85)
NPV (%)	94 (79–99)	89 (75–97)	100 (90–100)
PPV (%)	37 (19–58)	38 (18–62)	35 (15–59)
Diagnostic accuracy (%)	67 (54–79)	70 (57–82)	77 (64–87)
AUC*	0.73 (0.60–0.86)	0.69 (0.54–0.85)	0.87 (0.80–0.93)

Values are percentages; numbers in parentheses represent the 95% confidence intervals. AUC = area under the receiver operating characteristic curve; HR = high resolution; NPV = negative predictive value; PPV = positive predictive value UHR = ultra-high-resolution. * Subsequent statistical analyses indicated no significant differences of the per-patient AUC between the scanning modes: UHR vs. HR (*p* = 0.09), UHR vs. adjusted UHR (*p* = 1.00), adjusted UHR vs. HR (*p* = 0.07). Per-vessel analysis showed: UHR vs. HR (*p* = 0.68), UHR vs. adjusted UHR (*p* = 0.04), HR vs. adjusted UHR (*p* = 0.07)

## Discussion

This study evaluated the diagnostic performance of the HR mode, the UHR mode, and the adjusted for coronary imaging UHR mode of PCD-CT used in pre-TAVI scans for the detection of CAD with QCA as the reference standard. In the per-patient analysis, our findings indicate that both the UHR and adjusted UHR modes show high sensitivity and negative predictive value in this population with a very high calcium score. In the per-vessel analysis, the adjusted UHR mode maintains a high sensitivity and negative predictive value compared to the standard UHR and HR modes.

Our findings align with those of a prior study by Hagar et al., which reported a sensitivity, specificity, and accuracy of 96%, 84%, and 88%, respectively, per patient using the UHR mode for CAD detection, despite including distal segments in their analysis that we excluded in our study [12]. However, it is important to highlight the disparity in scan parameters between Hagar et al.‘s study and our standard and adjusted UHR mode. Their protocol involved a retrospective ECG-gated dual-source helical scan with a collimation of 120 mm × 0.2 mm, and tube voltage settings of 120 kV or 140 kV, along with automated tube current modulation and dose modulation with ECG pulsing between 20% and 80% of the R-R interval. In contrast, our study employed prospective ECG triggering with a tube voltage of 90 kV to minimize radiation exposure. As a result, the adjusted mode in our study achieved a lower radiation dose (CTDI_vol_ of 20.5 mGy [18.3–26.9] and dose length product of 306.5 mGy∙cm [262.5-419.8] in comparison to the threefold higher dose reported by Hagar et al. (CTDI_vol_ of 67.7 mGy ± 19.2 and dose length product of 936 mGy∙cm ± 278).

In our study, the implementation of the adjusted UHR scan mode was associated with a modest increased CTDI_vol_ compared to the standard UHR mode (standard: CTDI_vol_ 18.6 mGy [14.8–22.2], adjusted UHR mode: 20.5 [18.3–26.9]). However, the sensitivity at the per-patient level was similar, at 100%, across both modes. Nevertheless, at the per-vessel level, the sensitivity of the adjusted UHR was higher compared to the standard UHR mode, though the differences in diagnostic accuracy between the scan protocols were not statistically significant, for both the per-patient and per-vessel analyses. It is important to acknowledge that our study may have been underpowered to detect differences between the scan modes due to its small sample size (*n* = 20 per group).

Given these findings, the adjusted UHR protocol may be favoured for pre-TAVI scans in a clinical setting, as it offers improved sensitivity for CAD detection with only a modest increase in radiation dose, which is acceptable given the relatively older TAVI patient population. Future research with larger sample sizes is necessary to comprehensively compare the three scan protocols and assess whether the adjusted UHR protocol significantly enhances the diagnostic evaluation of CAD in pre-TAVI patients and therefore justifies the additional dose associated with this scan mode.

Furthermore, while the diagnostic performance of the HR mode was lower compared to the UHR and adjusted UHR modes, the SNR and CNR were significantly higher for the HR mode. Despite the higher SNR and CNR in the HR mode, these metrics do not always translate to better visual assessment. This paradox can be explained by the fact that, although human readers prioritise higher contrast over lower noise when assessing images, the overall diagnostic performance also heavily depends on resolution and the ability to detect small details. The UHR and adjusted UHR modes, with their superior resolution, can reveal more subtle diagnostic features that are crucial for accurate assessments, thus making them more effective despite potentially lower SNR and CNR.

A meta-analysis conducted by Gatti et al. demonstrated that implementing routine CCTA in the pre-TAVI workup could save 41% of ICAs if a disease prevalence of 40% is assumed [2]. In our study we had a similar disease prevalence of 40% and CCTA correctly ruled out CAD in 19/36 patients, indicating that 53% of the patients could have been safely deferred from ICA. However, the proportion of patients who could avoid invasive coronary angiography might be underestimated, as a recent meta-analysis by Aarts et al. showed no significant difference in clinical outcomes between patients with concomitant significant CAD who were treated with TAVI with and without preceding PCI at both short- and long-term follow-up [15].

Recently, the integration of spectral information with UHR mode has become available [16]. This development might improve the accuracy of PCD-CT for detecting CAD in pre-TAVI patients [16–18]. TAVI patients often have extensive coronary calcifications, leading to blooming artefacts and overestimation of stenosis, which may account for the high false positive rate and relatively low specificity for CCTA [3]. Incorporating spectral information with UHR imaging, particularly using optimal virtual mono-energetic imaging and calcium subtraction algorithms, may address this limitation, potentially improving the specificity of the pre-TAVI CT scan [19, 20].

There are some limitations regarding our study. At our institute, TAVI patients are typically scanned using the HR mode, resulting in a limited sample size of patients scanned on the adjusted and standard UHR mode for this study. Therefore, a larger sample size could make our findings more robust. Additionally, the presence of severe aortic stenosis in our patient cohort precluded the use of beta-blockers or nitro-glycerine. Furthermore, this study does not include a comparison of the diagnostic accuracy of PCD-CT with energy-integrating detector CT. Moreover, in our study, we reduced the tube potential to 90 kV in the adjusted UHR mode to limit radiation dose. However, this reduction comes with a trade-off: the lower tube potential increases the risk of blooming artefacts, particularly in patients with severe calcifications or stents, which could compromise image quality and diagnostic accuracy for detecting CAD. Increasing the tube potential would improve image quality, but it would also lead to a higher radiation dose. This highlights the challenge of finding a balance between dose reduction and image quality in patients with high coronary calcium scores or stents. Lastly, our analysis was limited to assessing the degree of stenosis and did not include plaque characterization.

In conclusion, while both UHR and adjusted UHR modes of PCD-CT show potential for improved sensitivity and negative predictive value for the detection of CAD in patients undergoing pre-TAVI scans, no definitive difference from HR mode was observed.

## Data Availability

No datasets were generated or analysed during the current study.

## Abbreviations

CAD: Coronary artery disease
CCTA: Coronary computed tomography angiography
CNR: Contrast-to-noise ratio
CTDI_vol_: Volume computed tomography dose index
HR: High-resolution
PCD: Photon-counting detector
QCA: Quantitative coronary angiography
SNR: Signal-to-noise ratio
STD: Standard deviation
TAVI: Transcatheter aortic valve implantation
UHR: Ultra-high-resolution
